# Metoprolol and bisoprolol ameliorate hypertrophy of neonatal rat cardiomyocytes induced by high glucose via the PKC/NF-κB/c-fos signaling pathway

**DOI:** 10.3892/etm.2022.11134

**Published:** 2022-01-10

**Authors:** Min Wang, Qingbo Lv, Liding Zhao, Yao Wang, Yi Luan, Zhengwei Li, Guosheng Fu, Wenbin Zhang

Exp Ther Med 19:871-882, 2020; DOI: 10.3892/etm.2019.8312

After the publication of the above article, the authors have realized that the β-actin and GAPDH bands featured in Figs. 3 and 5 on p. 876 and 877 respectively were selected incorrectly.

The authors have examined their original data, and have been able to identify the data that should have been used for both these figures. The corrected versions of Figs. 3 and 5 are shown opposite. Note that the revised control data shown for these Figures do not affect the overall conclusions reported in the paper. The authors apologize to the Editor of *Experimental and Therapeutic Medicine* and to the readership for any inconvenience caused.

## Figures and Tables

**Figure 3:**
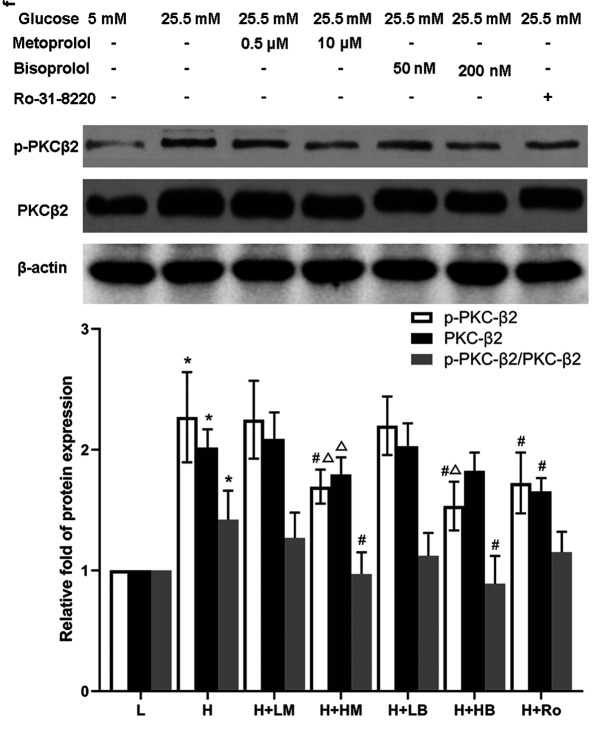
High dose of metoprolol decrease the expression and activity of PKC-β2 and high dose of bisoprolol decrease the activation of PKC-β2 in cardiomyocytes cultured in high glucose. p-PKC-β and total PKCβ protein levels were detected in cells. n=4–5. ^*^P<0.05 vs. L group, ^#^P<0.05 vs. H group, ^Δ^P<0.05 vs. corresponding low dose group of metoprolol or bisoprolol. L, low glucose; H, high glucose; LM, low metoprolol dose; HM, high metoprolol dose; LB, low bisoprolol dose; HB, high bisoprolol dose; Ro, Ro-31-8220.

**Figure 5:**
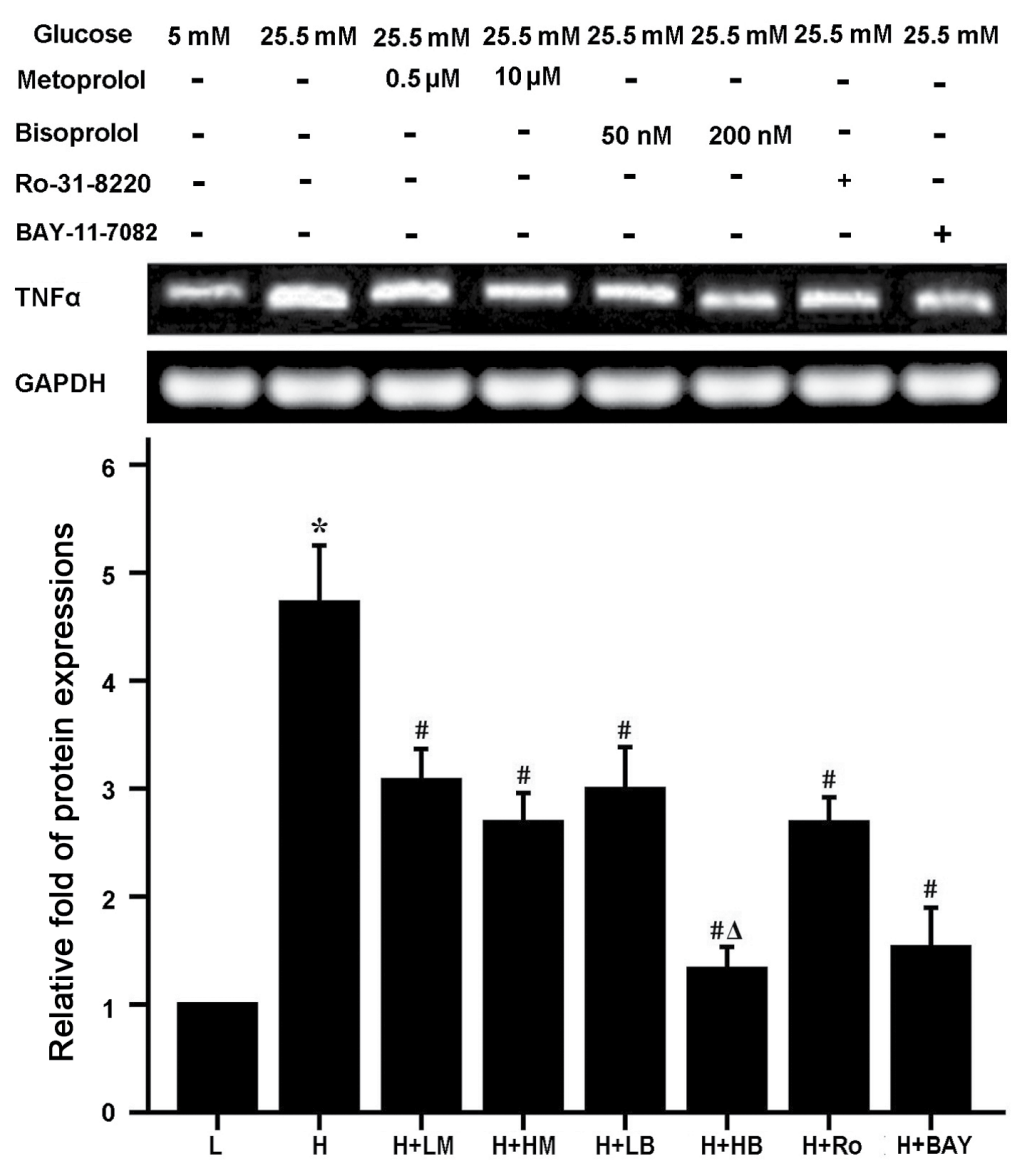
Effects of metoprolol, bisoprolol, PKC inhibitor Ro-31-8220 and NF-κB inhibitor BAY11-7082 on TNF-α expression in cardiomyocytes cultured in HG. n=4-5. ^*^P<0.05 vs. L group, ^#^P<0.05 vs. H group, ^Δ^P<0.05 vs. HG+LB group. L, low glucose; H, high glucose; LM, low metoprolol dose; HM, high metoprolol dose; LB, low bisoprolol dose; HB, high bisoprolol dose; Ro, Ro-31-8220; BAY, BAY11-7082; TNF-α, tumor necrosis factor-α.

